# Multiple Bands of Myocardial Bridging Involving the Left Anterior Descending and Posterior Descending Arteries: A Case Report and Its Clinical Implications

**DOI:** 10.7759/cureus.85938

**Published:** 2025-06-13

**Authors:** Kamal A Abouzaid, Ahmad Imam, Amira Basyouny, Vincent Ooi, Michael K Coffin

**Affiliations:** 1 Department of Anatomical Sciences, William Carey University College of Osteopathic Medicine, Hattiesburg, USA; 2 Department of Anatomical Sciences, Cairo University, Cairo, EGY; 3 Department of Dermatology, Cairo University, Cairo, EGY; 4 Department of Family Medicine, Forrest General Hospital, Hattiesburg, USA; 5 Department of Osteopathic Medicine, William Carey University College of Osteopathic Medicine, Hattiesburg, USA

**Keywords:** cardiac pacemaker, clinical and functional anatomy, left anterior descending artery, myocardial bridging (mb), posterior descending artery, rare variant

## Abstract

Myocardial bridging (MB) is a congenital coronary anomaly in which a segment of a coronary artery tunnels through the myocardium, potentially leading to ischemia, arrhythmias, or other clinical complications. While typically benign and most often involving a single band over the left anterior descending (LAD) artery, multiple myocardial bridges and involvement of other arteries, such as the posterior descending artery (PDA), represent rare anatomical variations.

During a routine dissection at the William Carey University College of Osteopathic Medicine, multiple MB bands were observed in a 65-year-old male cadaver. Three distinct, thin myocardial bridges were identified over the LAD artery. Additionally, a single, thick myocardial bridge was found over the PDA. Notably, the LAD displayed a unique course, turning onto the inferior surface of the heart. The coronary circulation was right-dominant, and a pacemaker was present, with leads extending to the right atrium and right ventricle. No other structural abnormalities were noted.

This case aligns with literature documenting variable MB presentations but stands out due to the coexistence of multiple bridges over the LAD and a separate, deep bridge over the PDA. These anatomical features may have contributed to the need for pacemaker placement, suggesting a history of conduction system dysfunction. The findings support previous observations that MB can be associated with arrhythmias and may complicate coronary blood flow.

Diagnostic and therapeutic strategies vary widely depending on the extent and functional impact of MB. Awareness of such variations is crucial for accurate diagnosis, risk assessment, and interventional planning in cardiology and cardiothoracic surgery.

## Introduction

The development of the human heart follows a complex series of embryological stages, beginning during gastrulation toward the end of the second week of gestation. A key component of cardiac development is the formation of the coronary arteries, which originate from the outermost layer of the heart, the epicardium. The epicardium itself derives from a specialized structure known as the proepicardium, a cluster of mesothelial cells located on the surface of the septum transversum. Under the influence of signals from the adjacent myocardium, some epicardial cells transition into mesenchymal cells, which further differentiate into the inner lining and smooth muscle layers of the coronary arteries. Additionally, neural crest cells contribute to the formation of the smooth muscle layers of the proximal coronary arteries. The connection between the coronary arteries and the aorta is established through the invasion of coronary endothelial cells into the aorta [1].

Coronary arteries typically traverse the subepicardial layer, between the epicardium and the myocardium. When myocardial fibers partially or entirely cover a coronary artery, this phenomenon is known as myocardial bridging (MB). In this condition, the overlying muscle band is referred to as the myocardial bridge, while the segment of the artery embedded within the myocardium is termed the “tunneled artery.” Although MB is generally considered benign, it can present diagnostic and therapeutic challenges in symptomatic patients [2].

The genetic and developmental factors responsible for coronary artery anomalies remain poorly understood due to limited research [3]. Pérez-Pomares et al. hypothesized that one possible explanation for the development of anomalies such as myocardial bridges is abnormal interaction between the myocardium and coronary vessels. This is seen in certain genetic mutants that exhibit disrupted cell polarity [4]. Irregularities in the distribution of myocardial growth factors or signaling molecules across the heart wall from the epicardium to the endocardium may also influence ventricular wall development and alter the precise positioning of embryonic coronary vessels relative to the ventricular cavity [4,5].

A meta-analysis by Hostiuc et al. estimated the overall prevalence of MB to be 19%. Prevalence varied significantly by diagnostic modality: autopsy studies identified MB in 42% of cases, computed tomography (CT) scans in 22%, and coronary angiography (CAG) in only 6%. The left anterior descending (LAD) artery was the most commonly affected vessel, involved in 82% of cases. Other frequently affected vessels included the diagonal, right coronary, marginal, and circumflex arteries. The authors concluded that autopsy remains the most reliable method for determining the true prevalence of myocardial bridges, while in clinical settings, high-resolution CT scanning offers superior diagnostic capabilities compared to CAG [6].

In this case report, we present a rare anatomical variation characterized by three distinct MBs on the LAD artery and an additional posterior bridge on the posterior descending artery (PDA). Through detailed anatomical observation and photographic documentation, this report contributes to the growing body of literature on MB and emphasizes the importance of recognizing its morphological diversity, including the potential for multiple coexisting bridges.

## Case presentation

In this report, we present a case of multiple MB bands observed during a pedagogical dissection of the external features and blood vessels of the heart, conducted as part of a first-year medical student anatomy course at William Carey University College of Osteopathic Medicine, Hattiesburg, MS, USA. The cadaveric donor was a 65-year-old Caucasian male, obtained through the University of South Alabama Anatomical Gift Program. The cause of death, as stated on the death certificate, was unknown.

**Figure 1:**
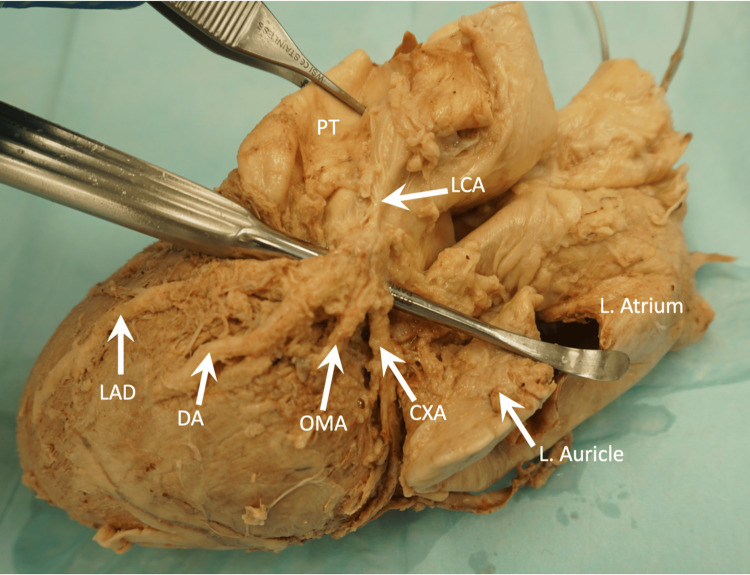
Left lateral view of the heart showing the branching pattern of the left coronary artery. CXA, circumflex artery; DA, diagonal artery; LAD, left anterior descending artery; L. atrium, left atrium; L. auricle, left auricle; LCA, left coronary artery; OMA, obtuse marginal artery; PT, pulmonary trunk

The left coronary artery (LCA) originated from the left aortic sinus and coursed between the ascending aorta and the left auricle. At the coronary sulcus, the LCA quadrifurcated into the LAD artery, diagonal artery (DA), obtuse marginal artery (OMA), and circumflex artery (CX). The LAD artery followed the anterior interventricular groove, descending toward the acute margin of the heart before turning and briefly running along the inferior surface of the heart, in the posterior interventricular groove. The DA traveled along the anterior surface of the left ventricle for 49 mm before dipping deeply into the myocardium. The OMA followed its typical course along the obtuse (left) margin of the heart. The CX artery traversed the coronary sulcus for a short distance before terminating in the myocardium, without anastomosing with the right coronary artery (RCA) (Figure 1).

**Figure 2:**
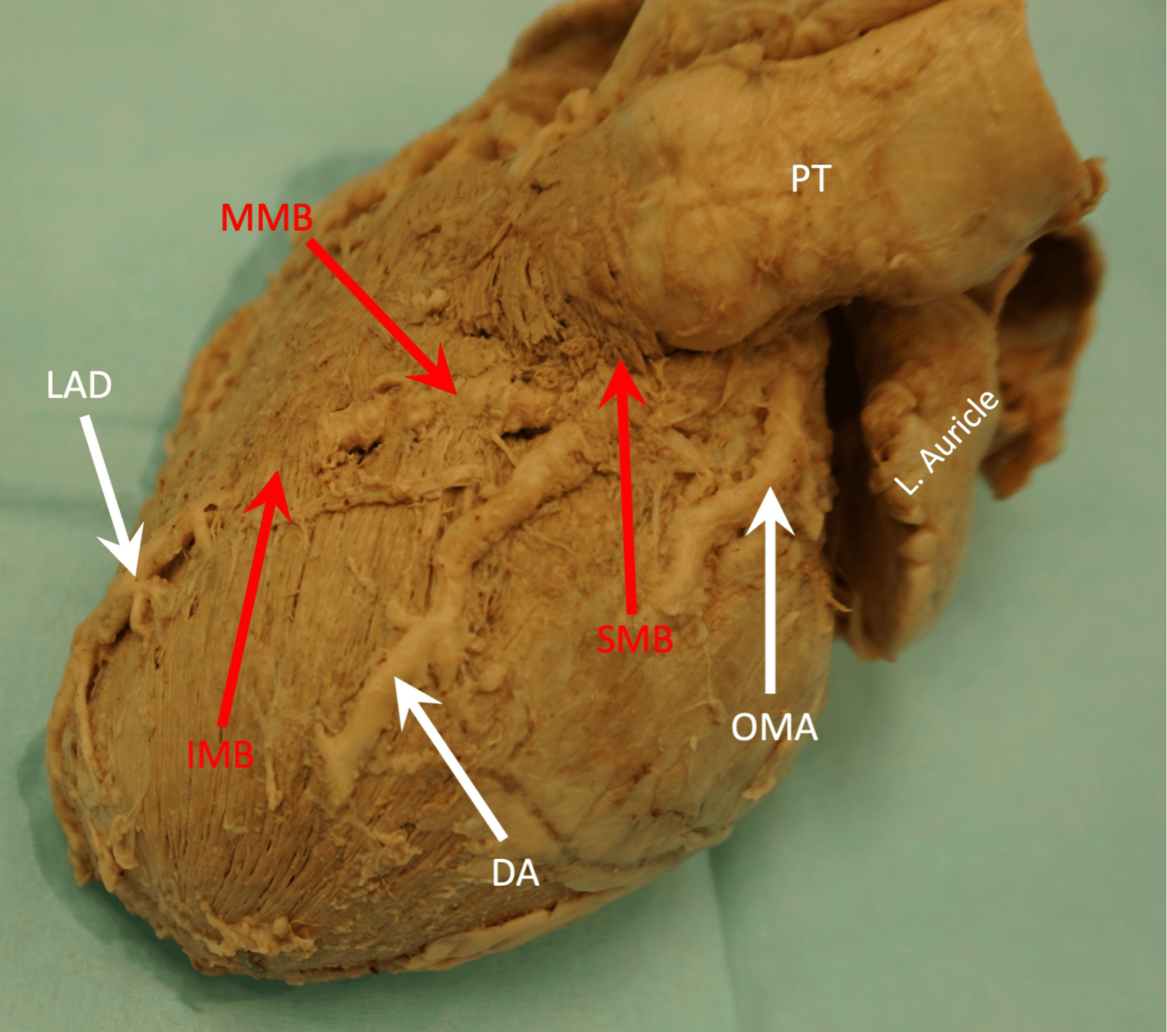
Superior view of the left side of the heart illustrating three myocardial bridging bands crossing the LAD artery. DA, diagonal artery; IMB, inferior myocardial bridging; L. auricle, left auricle; LAD, left anterior descending; MMB, middle myocardial bridging; OMA, obtuse marginal artery; PT, pulmonary trunk; SMB, superior myocardial bridging

**Figure 3:**
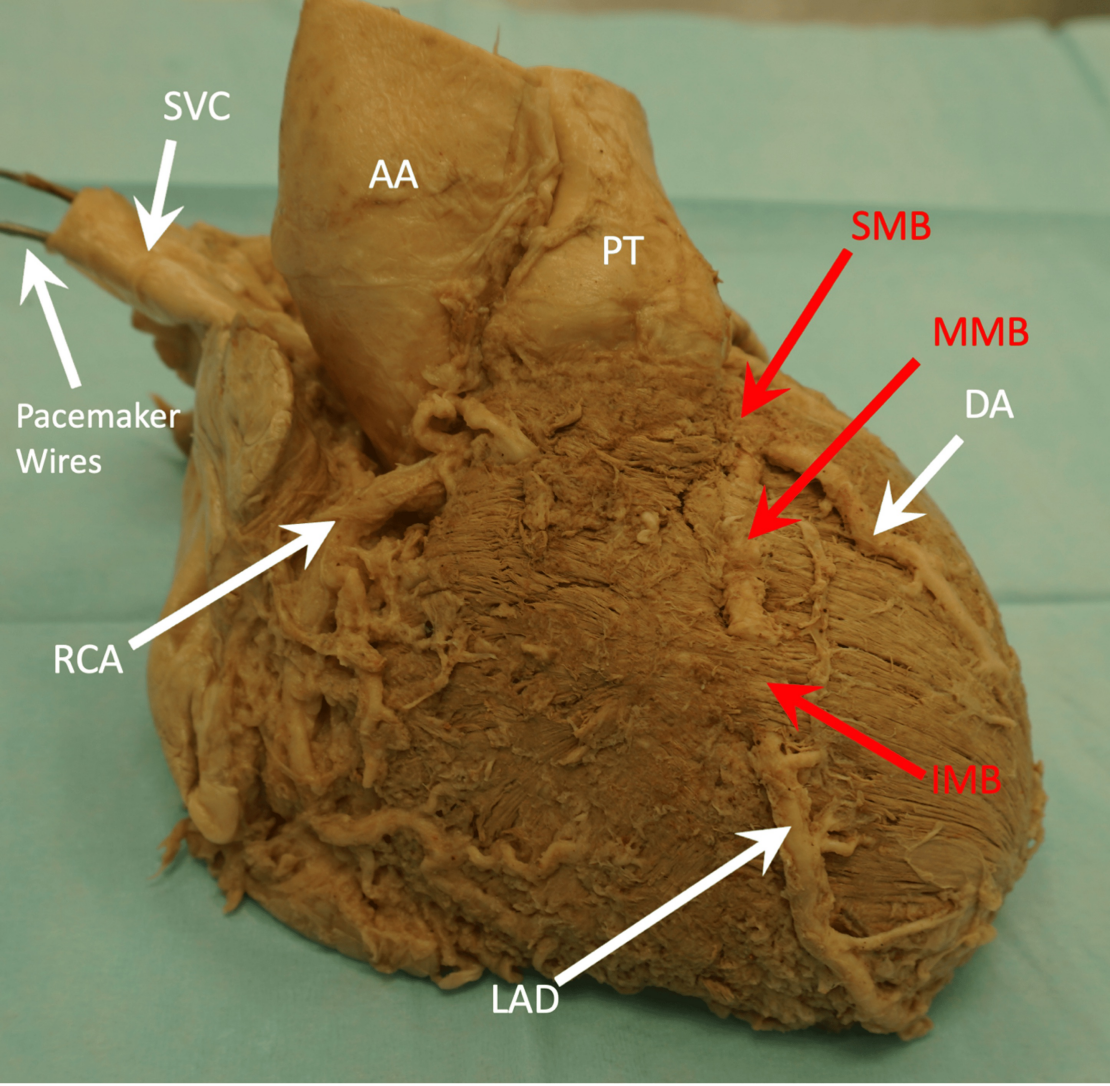
Anterior view of the heart displaying the LAD artery and the three myocardial bridging bands. AA, ascending aorta; DA, diagonal artery; IMB, inferior myocardial bridging; LAD, left anterior descending; MMB, middle myocardial bridging; PT, pulmonary trunk; RCA, right coronary artery; SMB, superior myocardial bridging

Three MB bands were noted. The superior MB band crossed over both the LAD and DA arteries and measured 10 mm in length. The middle MB band passed over the LAD and measured 8 mm in length, while the inferior MB band also crossed over the LAD and measured 11 mm in length (Figures 2, 3). All three bands were thin and consisted of a single layer of muscle fibers visible upon dissection.

**Figure 4:**
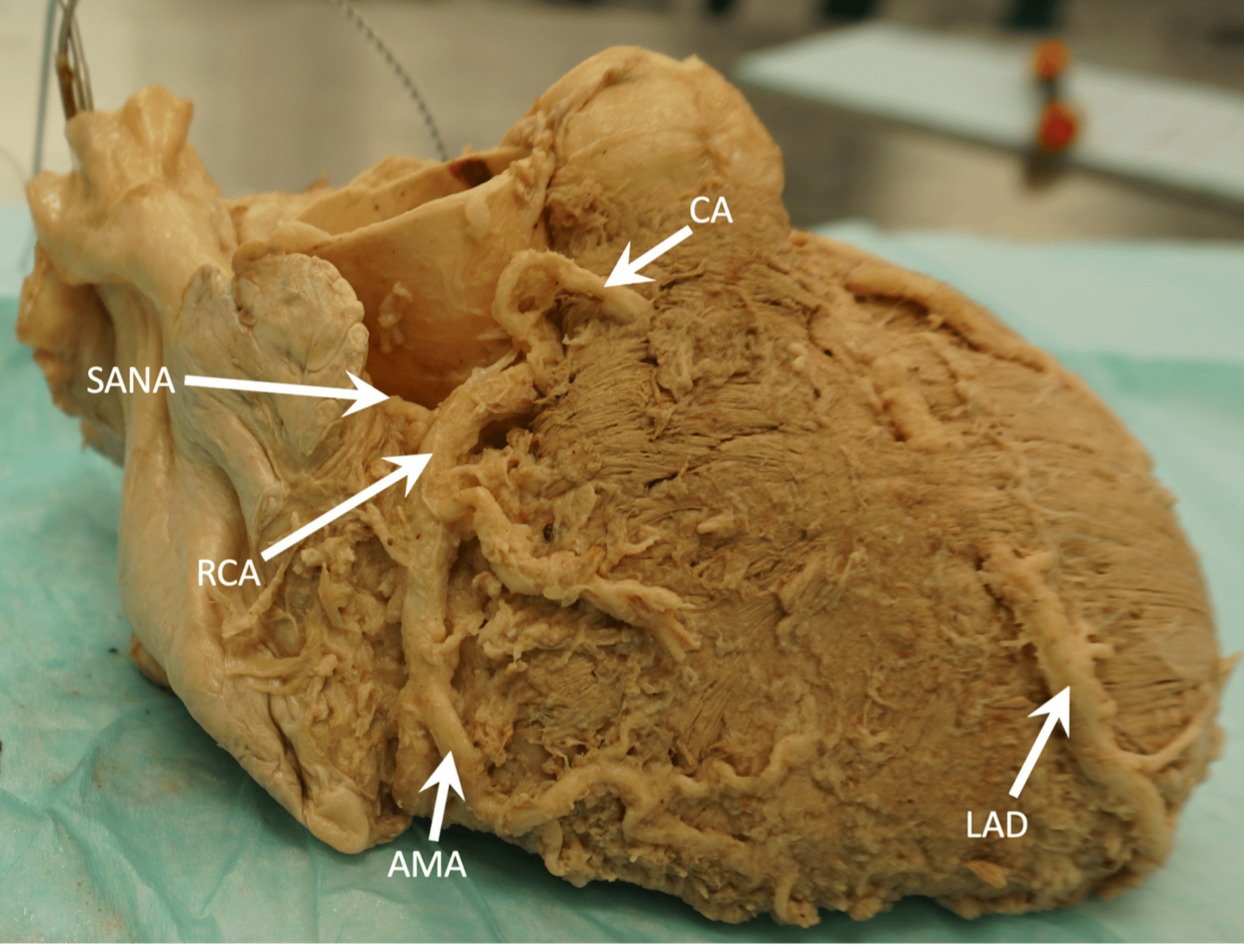
Right lateral view showing the RCA and its branches. AMA, acute marginal artery; CA, conal branch; LAD, left anterior descending; RCA, right coronary artery; SANA, sinoatrial nodal artery

The RCA arose from the right aortic sinus and traveled between the ascending aorta and the right auricle, reaching the coronary sulcus. The RCA then descended towards the acute margin of the heart and gave off several branches, including the conal artery, sinoatrial nodal artery (SANA), anterior atrial artery, anterior ventricular artery, and acute marginal arteries (Figure 4).

**Figure 5:**
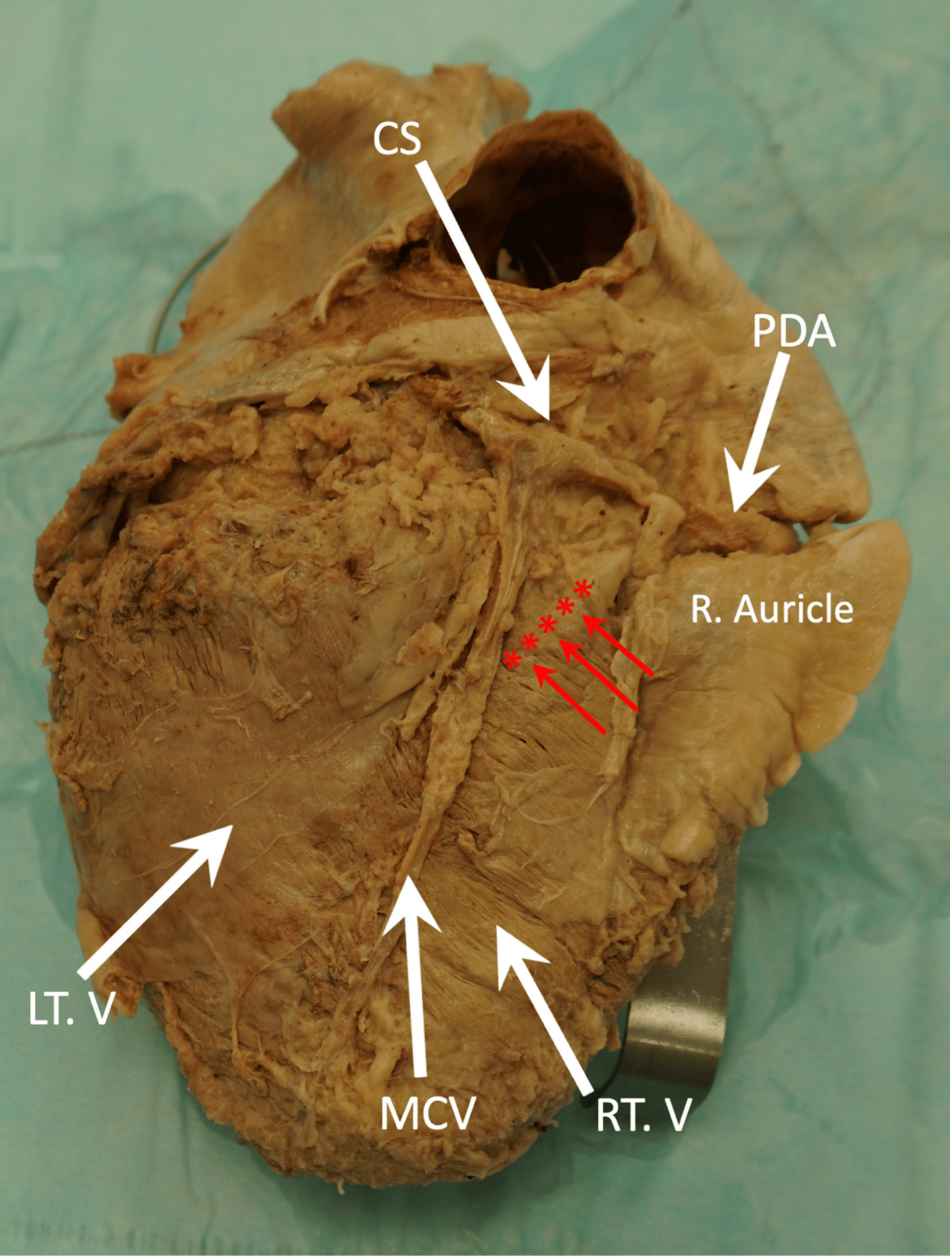
Inferior view of the heart (diaphragmatic surface) showing the PDA partially covered by a thick myocardial bridging band. CS, coronary venous sinus; LT. V, left ventricle; MCV, middle cardiac vein; PDA, posterior descending artery; R. auricle, right auricle; RT. V, right ventricle Red asterisks and arrows indicate the posterior myocardial band.

The RCA then turned along the diaphragmatic surface for 20 mm, giving off the atrioventricular nodal artery (AVNA), before continuing as the PDA. At this point, a distinct, thick MB band was observed crossing over the PDA (Figure 5). This band measured 22 mm in length and 2 mm in thickness. Distal to this bridging band, the PDA dipped deeply into the interventricular septum.

The middle cardiac vein (MCV) and the terminal portion of the left anterior descending artery (LAD) were observed in the distal part of the posterior interventricular sulcus (Figure 5). Although the PDA was not observed in the posterior interventricular sulcus, it is speculated that it maintained its territorial distribution, leading us to consider this heart's coronary circulation as right-dominant.

An additional finding that may be associated with MB was the presence of a pacemaker, connected to the heart by two wires (Figure 3). The tip of one wire was found in the right auricle, while the tip of the second wire was located in the right ventricle, in contact with the interventricular septum. No other abnormalities were observed, and the overall condition of the heart and its valves appeared normal.

## Discussion

MB is a well-documented congenital coronary anomaly in which a segment of a coronary artery is covered by myocardial tissue, potentially leading to a variety of clinical manifestations, including ischemia and arrhythmias. In our case, we observed three distinct, thin myocardial bridges over the LAD artery. Additionally, a single, thick myocardial bridge was found over the PDA.

In an autopsy study, researchers examined the anatomy of MB bands in 90 hearts from individuals ranging in age from stillbirth to 84 years, all with no known history of heart disease or cardiac-related death. MB bands were found in 50 hearts (55.6%). Of these, 35 hearts exhibited a single bridge affecting the LAD artery, 10 had two bridges, and 5 had three bridges. The MB bands were classified as superficial or deep. Deep MB bands were less common and featured longer muscle bundles compared to the superficial form [7]. This description aligns with the findings in our case, where three superficial MB bands were observed on the LAD, and a single, deeper, and longer band was noted on the PDA.

Murtaza et al. summarized the pathophysiological mechanisms that can lead to various clinical manifestations in MB cases. For instance, MB may result in a supply and demand imbalance, leading to diastolic flow restriction. It may also promote accelerated atherosclerosis through endothelial damage, distortion of the coronary artery at the myocardial bridge’s angle, and increased plaque formation near the bridge’s entrance. Lastly, MB can cause a branch steal effect, in which reduced perfusion pressure results from pressure loss within the bridged segment [8].

While MB is often asymptomatic, it can lead to significant clinical problems in some individuals. For example, Lee et al. noted that MB causes systolic compression that is detectable in fewer than 10% of cases via angiography [9]. However, the clinical outcome of MB can be serious, potentially causing myocardial ischemia in individuals without typical cardiac risk factors [8]. One report described the case of a 56-year-old male who developed acute ST-elevation myocardial infarction triggered by MB [10]. In a retrospective study, 15 of 81 individuals with MB exhibited signs of typical angina [11].

Furthermore, MB has been linked to the development of arrhythmias and heart block, which may explain the presence of a pacemaker in our case study. While the SANA and AVNA appeared unaffected, it is possible that the atrioventricular bundle and bundle branches were compromised, resulting in arrhythmias. This is likely due to impaired blood supply to the interventricular septum, which is typically perfused by the LAD and PDA. Faruqui et al. observed severe MB in two cases over the LAD, affecting perfusion to its territory and leading to ventricular fibrillation and supraventricular tachycardia [12]. Similarly, Feld et al. reported severe LAD MB on coronary angiogram in a patient with exercise-induced ventricular tachycardia [13], while Den Dulk et al. documented a case of LAD MB presenting with a paroxysmal atrioventricular block on exercise, as shown on CAG [14].

There is no single definitive test for diagnosing MB. Various diagnostic methods exist, each with differing levels of accuracy and limitations such as availability, contrast use, and radiation exposure. Some tests are better suited for anatomical evaluation, while others assess the functional consequences of MB [8]. Cardiac computed tomography angiography (CCTA) offers high spatial resolution images of the coronary arteries, enabling the detection of morphological characteristics of MB. However, while CCTA can depict MB, it cannot evaluate the functional significance of the bridged segments. CAG, particularly using multiple views to demonstrate maximal systolic narrowing, is a straightforward invasive technique for assessing MB [8]. Intravascular ultrasound (IVUS) has also been used to visualize coronary segments in patients with angiographic evidence of systolic vessel compression in the LAD. The bridged segment undergoes characteristic changes in cross-sectional shape during the cardiac cycle. Systolic compression of MB causes luminal reduction and produces the characteristic echolucent “half-moon” phenomenon on IVUS [15]. To assess the hemodynamic impact of MB, functional evaluations may include exercise echocardiography or dobutamine myocardial perfusion imaging. More recently, intra-coronary pressure wire measurements, such as the instantaneous wave-free ratio (iFR), have been suggested as ideal for evaluating MB patients [8].

Surgical treatment for symptomatic MB includes procedures such as surgical unroofing (supra-arterial myotomy) and coronary artery bypass grafting (CABG). Supra-arterial myotomy is the preferred treatment for patients with isolated LAD MB. In cases where LAD MB coexists with other cardiac conditions, favorable outcomes can be achieved by combining supra-arterial myotomy with additional cardiac procedures. For cases involving coronary stenosis proximal to the MB, either CABG alone or CABG combined with supra-arterial myotomy can yield positive results. Additionally, in diffuse MB cases, CABG may be performed following myotomy [16]. Surgical unroofing has proven to be a safe and effective option for relieving angina and exercise-induced arrhythmias in patients unresponsive to medical therapy [17]. Cerrato et al. conducted a meta-analysis of 18 studies evaluating various treatment options for MB and found that surgery was more effective than stenting in patients who failed to respond to medical management [18].

## Conclusions

While MB is a well-documented congenital anomaly, the presence of multiple bands, particularly involving both the LAD and PDA, is an exceptional and clinically significant finding. This case offers valuable anatomical insights that could enhance clinical understanding and inform procedural planning in both interventional cardiology and cardiac surgery.
